# Shading decreases lodging resistance of wheat under different planting densities by altering lignin monomer composition of stems

**DOI:** 10.3389/fpls.2022.1056193

**Published:** 2022-11-17

**Authors:** Yongli Luo, Yonglan Chang, Chunhui Li, Yuanyuan Wang, Haixing Cui, Min Jin, Zhenlin Wang, Yong Li

**Affiliations:** State Key Laboratory of Crop Biology, College of Agronomy, Shandong Agricultural University, Tai’an, Shandong, China

**Keywords:** wheat, shading, stem, lignin, lodging

## Abstract

To clarify the influences of shading stress and planting density on the lignin monomer composition of wheat stems and their relationship with lodging resistance, Lodging resistant variety Shannong 23 (SN23) and lodging sensitive variety Shannong 16 (SN16) were grown during 2018−2019 and 2019−2020 growing seasons. The planting densities were 150 × 10^4^ plants ha^-1^ (D1), 225 × 10^4^ plants ha^-1^ (D2) and 300 × 10^4^ plants ha^-1^ (D3). At the jointing stage, an artificial shading shed was used to simulate shading stress. Then the effects of shading on stem morphological characteristics, lignin monomer composition and lodging resistance of wheat under different planting densities were studied. Results indicate that shading at the jointing stage increased the length of basal internodes and the plant height and moved the height of center of gravity (CG) upward. Moreover, the stem diameter and the wall thickness decreased by 0.10−0.53 mm and 0.18−0.40 mm, respectively. The stem filling degree was reduced accordingly. As indicated by the correlation analysis and the stepwise regression analysis, shading-induced lodging mainly resulted from changes in the stem morphological characteristics and lignin accumulation. The influential magnitude of these factors was ordered as follows: stem filling degree, wall thickness, lignin content, contents and proportions of monomers S and H, and length of the second internode. The expression abundance of *TaPAL*, *TaCOMT*, *TaCCR*, and *TaCAD* declined in response to shading stress and high planting density. As a result, the distribution ratios of photosynthetic carbon sources to lignin monomers S, G and H were changed. The lignin content of stems on the day 42 after the jointing stage decreased by 18.48%. The monomer S content decreased, while the content and proportion of monomer H increased, thus weakening the breaking strength of wheat stems.

## 1 Introduction

Wheat is the main grain crop in China. To ensure China’s food security, it is crucial to consistently raise the yield of wheat produced per unit of land area. In recent years, with the impact of global climate change, wheat has frequently encountered rainy and sunless weather during its growing season, indicating decreased solar radiation (Yang et al., 2022). According to reports, the total sunshine hours in the Huang-Huai-Hai wheat region of China have decreased by 3.74−9.22 hours annually on average. Furthermore, sunshine hours have reduced by 2.98−3.67 hours annually on average during the winter wheat growing season (Cai and Jiang, 2011). Low light stress dramatically affects the lodging resistance of wheat stems by reducing stem filling degree (Hussain et al., 2019). Increasing plant density to enhance spikes per unit area is the main cultivation measure to further improve the wheat yield. However, among the dense planting population, the light environment becomes worse, including the weakened light intensity and the reduced ratio of red light to far red light, and so on. As a result, shading avoidance response arises, such as increased stem elongation and decreased stem diameter (Pantazopoulou et al., 2021), resulting in the poor quality of stems and a high risk of lodging. Stem lodging has become a significant barrier to the improvement of yield and quality in wheat. Consequently, studying the mechanism of low light stress on stem lodging of wheat with different population structures has crucial theoretical and productive significance.

Stem lodging is the permanent displacement of stems in the vertical direction (Dixon et al., 2020). Lignin is an important raw material for secondary wall thickening. In the lignification of plant cell walls, lignin is synthesized in the cytoplasm and transported to the polysaccharide framework of the cell walls for polymerization and deposition. In this way, the hardness of the tissue can be enhanced and the support and pressure resistance of cells can be increased (Alejandro et al., 2010; Miao and Liu, 2010). Lignin is strongly related to the lodging resistance of crop stems (Hussain et al., 2020). The lignin content accounts for 10%−20% of gramineous crops (Ragauskas et al., 2014). Increasing lignin content results in higher mechanical strength of crop stems, while decreasing lignin content causes a higher lodging risk (Wang et al., 2015; Chen et al., 2018). Rice dwarf mutant pex that has high expression of lignin synthesis pathway genes and high lignin content in the stem owns strong lodging resistance (Ke et al., 2019).

Lignin is mainly polymerized by three monomers, syringyl lignin (S), guaiacyl lignin (G) and p-hydroxyphenyl lignin (H). When the total lignin content maintains constant, changes in lignin monomer composition have no significant effect on the morphology and development of plants (Vanholme et al., 2012). However, the contents and proportions of lignin monomers are closely related to the lodging resistance of plants (Li et al., 2017). The high monomer G content improves the lodging resistance of rice stems (Li et al., 2015). The S/G ratio is negatively correlated with lodging resistance (Wei et al., 2017). Zheng et al. (2017a) discovered that the decreased monomer S content, the decreased S/G ratio, and the increased monomer H content are the key causes for decreasing the breaking strength under high nitrogen fertilizer and high density. It was also found that monomer S can help improve the breaking strength of wheat stems, the dominant structural units. However, the high proportion of monomer H is not conducive to the breaking strength of wheat stems (Luo et al., 2019). Paeonia lactiflora mutants overexpressing *HCT* have higher breaking strength due to their increased lignin content and contents of monomers G and S (Zhao et al., 2021). To summarize, the findings on the relationship between lignin monomers composition and lodging resistance of crop stems are inconsistent, so the relationship needs to be further clarified. Therefore, it is necessary to further study the changes in lignin monomers composition in wheat stems in response to low light stress and its relationship with lodging resistance.

Plants synthesize lignin through a series of biochemical reactions, which require many enzymes (**Figure S1**). Phenylalanine ammonia lyase (PAL), cinnamate 4-hydroxylase (C4H), p-coumaroyl shikimate 3’-hydroxylase (C3’H), p-hydroxycinnamoyl CoA:shikimate/quinate hydroxycinnamoyltransfera (HCT), and 4-hydroxycinnamate:CoA ligase (4CL) are the key enzymes of carbon flow distribution in the lignin synthesis pathway. Downregulation of these genes can decrease the lignin content, change the lignin monomer composition, and thus weaken the rigidity of stems (Xu et al., 2011; Sykes et al., 2015; Liu et al., 2019; Fang et al., 2020). Ferulate/coniferaldehyde 5-hydroxylase (F5H), caffeate/5-hydroxyferulate 3-O-methyltransferase (COMT) and caffeoyl-CoA 3-O-methyltransferase (CCoAOMT) are the key enzymes in the formation of lignin monomers. Downregulation of these genes has no significant effect on total lignin content,but can change the composition of lignin monomers and thus decrease the breaking strength of stems (Wagner et al., 2011). Peroxidase (POD) and laccase (LAC) are key enzymes in the lignification process at later stages (Ruben et al., 2010; Fetherolf et al., 2020).

Lignin is a photosensitive phenolic compound. Changes in the light environment directly affect the lignin synthesis pathway. Due to shading stress, the expression abundance of *PAL*, *4CL*, *F5H*, and *LAC* dropped, reducing the lignin content in the tea plant (Teng et al., 2021). Low light stress reduced the expression of *C3’H*, *CCR*, *CCoAOMT*, and *POD* and decreased the activities of POD, CAD, 4CL and PAL, finally reducing the lignin content in soybean stems (Liu et al., 2018; Hussain et al., 2021). However, it is unclear about the mechanisms of lignin monomers composition in response to low light stress in wheat under different planting densities. Thus it is urgent to ascertain this mechanism in the study of lodging resistance and yield increase cultivation of wheat.

In summary, the effects of low light stress on lignin metabolism, and lignin monomers composition, as well as their relationships with lodging resistance are required to be further studied. Hence, in this experiment, low light stress at the jointing stage was simulated using artificial shading targeting two wheat varieties with significantly different lodging resistance in different planting densities. Ultimately, this study aims to explore a cultivation regulation way for increasing lodging resistance under the conditions of overcast rain and scant sunshine and guide for the breeding of wheat varieties with lodging resistance and shade tolerance.

## 2 Materials and methods

### 2.1 Experimental site

A field experiment was conducted at the agricultural experiment station of Shandong Agricultural University in Tai’an City, Shandong Province (36°9’N, 117°9’E; altitude 128 m) during the two wheat growing seasons of 2018−2019 and 2019−2020. This region where the experimental site is located has a warm temperate semi-humid continental monsoon climate. During the wheat growing season, it has solar radiation of 3141.33 MJ m^-2^, an average temperature of 8.92°C, and precipitation of 193.06 mm. The soil of the experimental site is sandy loam, on which corn was planted. All the corn stalks were returned to the field after harvest. Before sowing, the soil at a depth of 0−20 cm had organic matter content of 12.81** g** kg^-1^, total nitrogen was 1.08** g** kg^-1^, alkali hydrolyzable nitrogen of 87.3** g** kg^-1^, available phosphorus of 31.21** g** kg^-1^, and available potassium of 91.83** g** kg^-1^.

### 2.2 Experimental design

Two winter wheat varieties with significantly different lodging resistance, lodging sensitive variety Shannong 16 (SN16) and lodging resistant variety Shannong 23 (SN23), were planted in the main area. The sub area was shading treatment, with natural light (S0) as the control, including shading from the jointing stage to the heading stage (S1) and shading from the jointing stage to the maturity stage (S2). Three planting densities were set in the sub-sub area, which were 150 × 10^4^ plants ha^-1^ (D1), 225 × 10^4^ plants ha^-1^ (D2) and 300 × 10^4^ plants ha^-1^ (D3). The specific treatment methods were as follows: at the jointing stage (when the first basal internode of wheat elongates to 1.5−2 cm), a removable shading shed was built using a self-made scaffold and a black nylon net with light transmittance of 50% for shading treatment. The photosynthetic effective radiation (PAR) intensity at 1 m above ground from the jointing stage (April 5, 2020) to the maturity stage (June 9, 2020) of wheat under normal light and shading treatment was shown in **Figure S2**. Shading stress treatment significantly reduced the PAR intensity in the upper part of wheat canopy. In order to ensure the normal ventilation conditions, the height of the shed top was 2 m from the ground. Each process was repeated three times, and the area of each plot was 3 m × 3 m = 9 m^2^. Artificial furrow was used for planting and seedlings were fixated at the three leaf stage. The nitrogen fertilizer used was urea (46% N), with a total application amount of 240 kg ha^-1^. Specifically, 50% was used as base fertilizer, and the remaining 50% was used as topdressing at the jointing stage. Total application amounts of potassium chloride (60% K_2_O) and calcium superphosphate (12% P_2_O_5_) were 120 kg ha^-1^ and 150 kg ha^-1^, respectively. Both phosphorus and potassium fertilizers were applied as base fertilizers at one time. Other field management was the same as that of general high-yield fields. The sowing dates during wheat growing seasons were October 8, 2018 and October 12, 2019 and the corresponding and harvest dates were June 6, 2019 and June 9, 2020.

### 2.3 Determination items and methods

#### 2.3.1 Determination of light interception of wheat canopy

The SunScan (Delta, UK) canopy analyzer was adopted. At the anthesis stage (GS65), sunny and cloudless weather was chosen. From 10:00 to 13:00, the PAR in the upper part of the wheat canopy (10 cm above the spike) = and the PAR at the bottom (TPAR) in the lower part of the wheat canopy (10 cm from the ground) were measured. The canopy PAR interception rate (CaR) was calculated as follows: CaR(%) = (PAR-TPAR)/PAR × 100%.

#### 2.3.2 Determination of morphological characteristics and filling degree of wheat stems

At anthesis stage (GS65), milk stage (GS75) and dough stage (GS85) (Zadoks et al., 1974), the morphological indexes including plant height, internode length, height of center of gravity, stem diameter and wall thickness were measured.

Wheat stems filling degree was measured from the second basal internode with a length of 2 cm. At this time, it was recorded as day 0, and samples were taken every 7 days. Samples were oven-dried at 105°C for 30 min and then oven-dried at 50°C to constant weight. The dry weight per unit length was used to define the filling degree.

#### 2.3.3 Determination of breaking strength of wheat stems

At anthesis stage (GS65), milk stage (GS75), and dough stage (GS85), ten uniform stems were selected for each treatment, with their second basal internodes were cut. Then the YYD-1 strength tester (Zhejiang Top Instrument Co., Ltd.) was used to measure the breaking strength of stems according to previous studies (Peng et al., 2014).

#### 2.3.4 Determination of lignin content in wheat stems

The sampling method was the same as 3.3.2. Ten uniform stems were selected for each treatment. The second basal internodes were cut and quickly frozen in liquid nitrogen and then stored in a -80°C refrigerator for determination of lignin content according to previous studies (Zheng et al., 2017a). The lignin content was expressed by the absorbance value at 280 nm per milliliter of fresh sample per unit mass.

#### 2.3.5 Determination of lignin monomers in wheat stems

The sampling method was the same as 3.3.2. Twenty uniform stems were selected for each treatment. Samples were oven-dried at 105°C for 30** min** and then oven-dried at 50°C to constant weight. Lignin monomers were determined by alkaline nitrobenzene oxidation method (Zheng et al., 2017b). The qualitative and quantitative analysis of lignin monomers in wheat stems was carried out by ultra high performance liquid chromatography coupled with triple quadrupole mass spectrometry. The steps were as follows: 1.0** g** of sample was weighed. Add 30 mL of NaCl, absolute ethanol solution, 95% ethanol, acetone, chromatographic chloroform/chromatographic methanol to remove the pigment, protein, lipid and other components in the sample. The sediment was put into the oven for drying at 50°C and then was accurately weighed. 0.02** g** sediment was put into the digestion tube. 3 mL of 2 M NaOH and 0.5 ml of nitrobenzene was added into the tube for microwave digest at 170°C for 1 h. Then ethyl acetate was added for extraction after digestion. 7 ml of ethyl acetate phase was evaporated with a vacuum centrifuge concentrator (Thermo Fisher), 6 mL of 50% acetonitrile water was added for redissolution. Finally, the redissolved solution was filter through 0.22 µm organic system filter membrane for concentration determination.

#### 2.3.6 Determination of gene expression of key enzymes in the lignin synthesis pathway of wheat stems

Samples were taken on day 0 when the second basal internode of wheat had an approximate length of 2 cm. After that, samples were taken every 7 days until the end of the dough stage (GS85). Five uniform stems were selected for each treatment. The second basal internodes were cut and ground into powder in liquid nitrogen. Ribonucleic acid (RNA) was extracted using the plant sample RNA extraction kit manufactured by Tiangen Biotechnology (Beijing, China; Cat. No. DP432), according to manufacturer instructions. The PrimeScript™ RT reagent Kit (TaKaRa Bio, No. RR047B) with g-deoxyribonucleic acid (gDNA) eraser was used for cDNA reverse transcription. The target gene and internal control of each sample were subject to quantitative real-time PCR (qRT-PCR), and each sample was tested in three replicates. The relative expression levels were analyzed using the comparative 2^-△△CT^ method. The primer sequences for *TaPAL*, *TaCCR*, *TaCAD*, *TaCOMT* and *TaActin* are listed in **Table S1**.

### 2.4 Statistical analysis

The obtained experimental data were statistically analyzed using Excel 2007 and DPS 7.05. Differences between mean values were determined using the least significant difference (LSD) test at *P* < 0.05 and *P* < 0.01. All figures were drawn using the Sigmaplot 10.0.

## 3. Results and analysis

### 3.1 Effects of shading on PAR interception rate of wheat under different planting densities

It was demonstrated that SN16 had a higher PAR interception rate than SN23 (**Figure S3**). Under the same planting density, shading reduced the PAR interception rate of wheatin the order of S0 > S1 > S2. Under the same shading treatment, the trend was D3 > D2 > D1.

### 3.2 Effects of shading on the plant height, internode configuration and CG height of wheat under different planting densities

**Figure 1:**
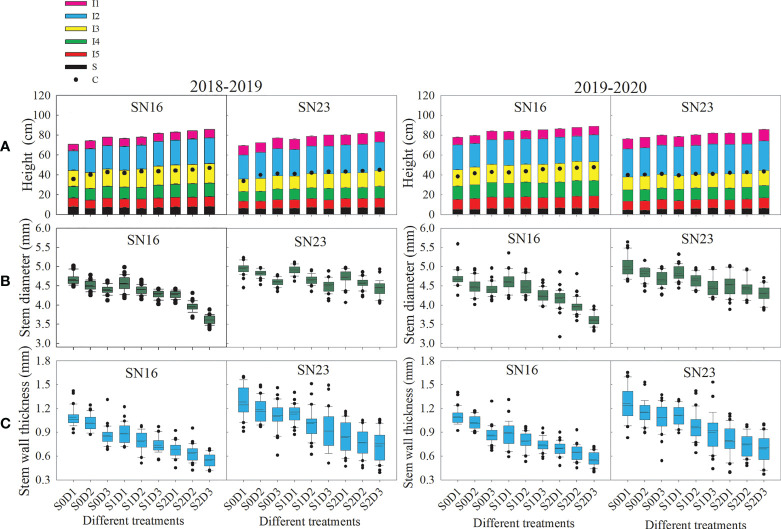
Effects of shading on morphological indexes stem of wheat under different planting densities. **(A)** Plant height, configuration of internnodes, and center of gravity of wheat at milk stage. Error bars represent the standard deviation of the mean (n = 10). **(B, C)** Stem diameter and wall thickness of the second basal internode. Solid lines and dotted line indicate medians and means, respectively. Box boundaries indicate upper and lower quartiles, whisker caps and points indicate internal limiting and outliers (n = 30).

The plant height, internode length and CG height (at the milk stage) of two wheat varieties are shown in **Figure 1A**. Compared with lodging sensitive wheat SN16, lodging resistant wheat SN23 had low values in plant height, the length of the 1^st^-4^th^ basal internodes, and the CG height but higher values in the length of the 5^th^ basal internode and spike length. With an increase in the planting density, the internode length, spike length, plant height, and CG height of two wheat varieties increased. When planting density was increased by 75 × 10^4^ plants ha^-1^, the plant height and CG height increased by 1.82 cm and 1.11 cm, respectively under shading from the jointing stage to the heading stage (S1), and increased by 1.57 cm and 0.86 cm, respectively under shading from the jointing stage to the maturity stage (S2).

Shading had significant effects on plant height, internode configuration and CG height of two wheat varieties. Under the same planting density, shading significantly increased plant height, CG height, length of the 2^nd^-5^th^ internodes (especially the 4^th^ internode), and spike length. Moreover, with the extension of shading time, these indices significantly increased. As for the two wheat varieties in 2018−2019 wheat growing seasons, samples under treatments S1D1 and S2D1 had the average plant height increased by 8.88% and 19.35% and the average CG height increased by 16.17% and 26.01%, compared with those under treatment S0D1. Additionally, samples under S1D2 and S2D2 treatments had the average plant height increased by 7.17% and 13.23% and the average CG height increased by 6.74% and 11.22%, compared with those under treatment S0D2. Samples under treatments S1D3 and S2D3 had the average plant height increased by 4.51% and 9.20% and the average CG height increased by 3.55% and 9.37%, compared with those under treatment S0D3. The results suggested that the changing range of shading stress affecting the plant height and CG height of two wheat varieties decreased with increased density, which might be due to shade avoidance response (**Figure 1A**).

### 3.3 Effects of shading on the stem diameter and wall thickness of wheat under different planting densities

The stem diameter and wall thickness of the second basal internode were measured at the anthesis, milk, and dough stages. The results show that lodging resistant variety SN23 had much larger values than the lodging sensitive variety SN16 (**Figures 1B, C**). With increasing planting density, the stem diameter and wall thickness of the second basal internode of wheat decreased significantly. When planting density was increased by 75 × 10^4^ plants ha^-1^, samples under S0, S1, and S2 treatments had the average stem diameter decreased by 0.15 mm, 0.18 mm and 0.21 mm, and the average wall thickness reduced by 0.10 mm, 0.09 mm and 0.06 mm, respectively, under S0, S1, and S2 treatments.

Taken 2018−2019 wheat season as an example, compared with those under S0D1 treatment, the stem diameter of two wheat varieties was increased by 0.10 mm and 0.41 mm on average, and thickness increased by 0.18 mm and 0.43 mm on average, respectively, under S1D1 and S2D1 treatments. Additionally, compared with those under S0D2 treatment, the stem diameter of two wheat varieties was increased by 0.12 mm and 0.42 mm on average, and thickness increased by 0.20 mm and 0.40 mm on average, respectively, under S1D2 and S2D2 treatments. Compared with those under S0D3 treatment, the stem diameter of two wheat varieties was increased by 0.15 mm and 0.53 mm on average, and thickness increased by 0.16 mm and 0.35 mm on average, respectively, under S1D3 and S2D3 treatments (**Figures 1B, C**).

### 3.4 Effects of shading on the filling degree of the second basal internode in wheat under different planting densities

**Figure 2:**
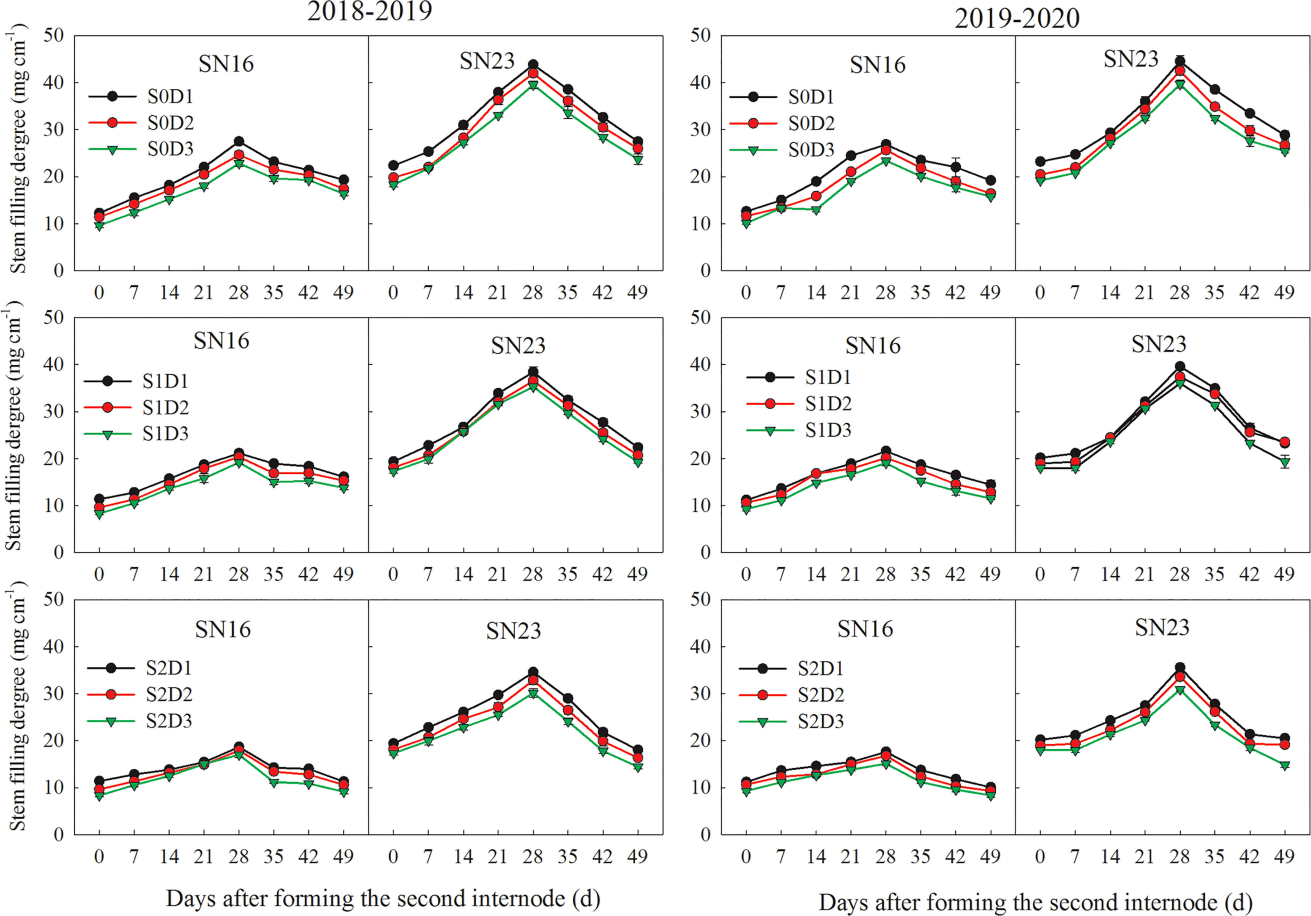
Effects of shading on filling degree of the second basal internode of wheat under different planting densities. Error bars represent the standard deviation of the mean (n = 10).

With the progress of growth, the filling degree of wheat stems first increased, then decreased, and finally peaked on day 28 after the formation of the second basal internode (**Figure 2**). Lodging resistant wheat SN23 had a significantly higher filling degree than lodging sensitive wheat SN16 in the second basal internode. Shading and planting density had significant negative effects on the stem filling degree of two wheat varieties. With increasing planting density, the second basal internode filling degree of wheat was decreased significantly.

When planting density was increased by 75 × 10^4^ plants ha^-1^, the average stem filling degree of samples under S0, S1, and S2 treatments was decreased by 1.93 mg cm^-1^, 1.33 mg cm^-1^, and 1.44 mg cm^-1^, respectively. As for the two wheat varieties in 2018-2019 wheat season, samples under treatments S1D1 and S2D1 had the average stem filling degree increased by 4.02 mg cm^-1^ and 6.89 mg cm^-1^, compared with those under treatment S0D1. Additionally, samples under S1D2 and S2D2 treatments had the average stem filling degree increased by 3.16 mg cm^-1^ and 6.17 mg cm^-1^, compared with those under treatment S0D2. Samples under treatments S1D3 and S2D3 had the average stem filling degree increased by 2.82 mg cm^-1^ and 5.91 mg cm^-1^, compared with those under treatment S0D3 (**Figure 2**).

### 3.5 Effects of shading on the breaking strength of wheat stems under different planting densities

**Figure 3:**
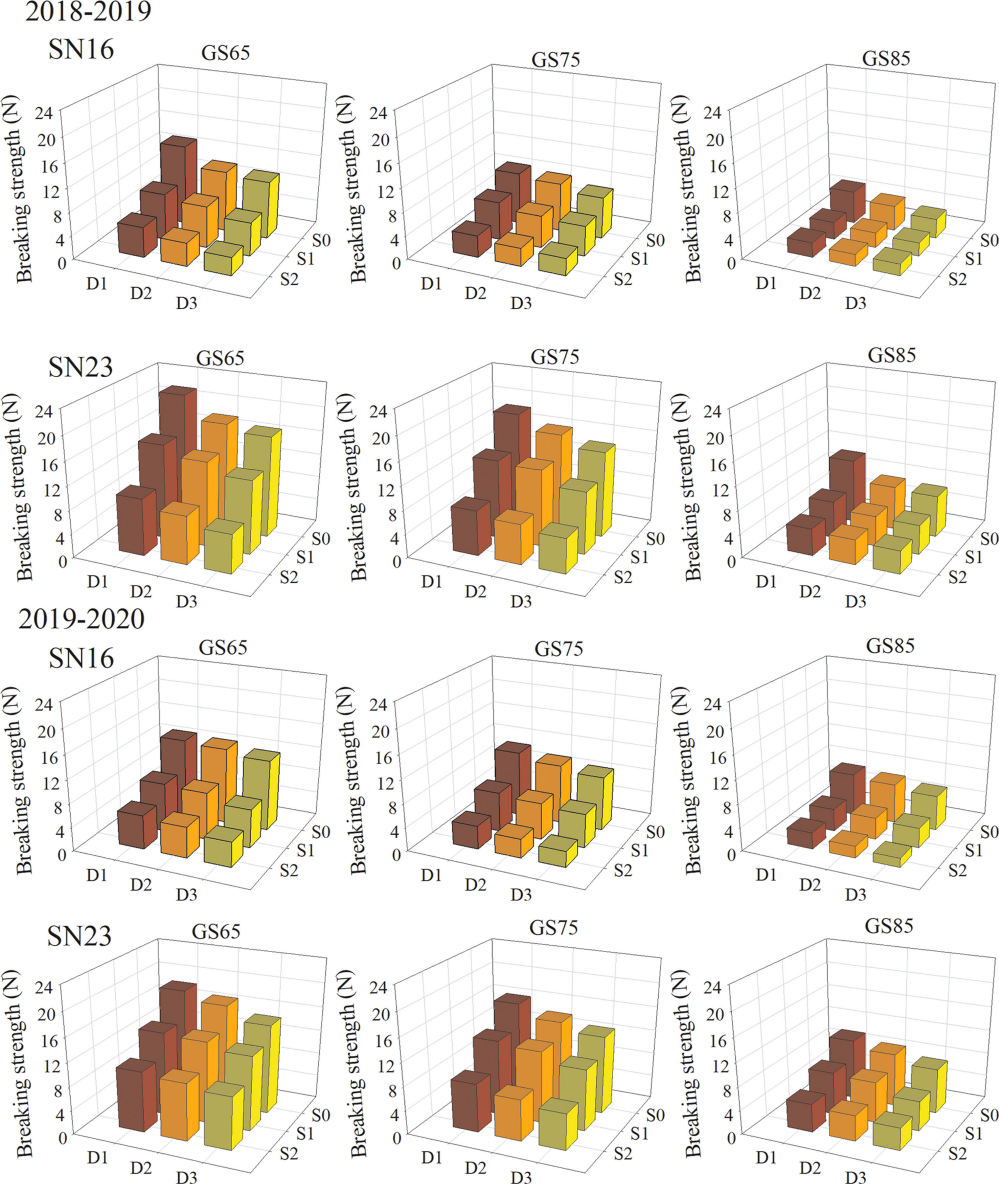
Effects of shading on breaking strength in different winter wheat populations at anthesis stage.

In field production, the lodging of wheat stems usually occurs in the middle and late stages. Therefore, the breaking strength of the second basal internode was measured at the anthesis stage (GS65), milk stage (GS75) and dough stage (GS85) of wheat in 2018−2019 and 2019−2020. The results show that the lodging sensitive variety SN16 had significantly weak breaking strength than lodging resistant variety SN23 (**Figure 3**). With the process of growth, the second basal internode of the two wheat varieties had gradually decreased breaking strength, shown as GS65 > GS75 > GS85. The breaking strength of the second basal internode of two wheat varieties was decreased with increased planting density at different stages, shown as D1 > D2 > D3. Shading treatment significantly reduced the breaking strength of stems, and with the extension of shading time, the declined range of stem breaking strength was increased. As for the two wheat varieties in 2018−2019 wheat season, samples under treatments S1D1 and S2D1 had the average breaking strength increased by 34.47% and 58.49%, compared with those under treatment S0D1. Additionally, samples under S1D2 and S2D2 treatments had the average breaking strength increased by 28.21% and 57.16%, compared with those under treatment S0D2. Samples under treatments S1D3 and S2D3 had the average breaking strength increased by 31.26% and 58.84%, compared with those under treatment S0D3 (**Figure 3**). Taken 2018−2019 wheat season as an example, compared with that under S0D1 treatment, breaking strength of two wheat varieties was increased by 34.47%, and 58.49% on average, respectively, under S1D1 and S2D1 treatments. Additionally, compared with that under S0D2 treatment, breaking strength of two wheat varieties was increased by 28.21%, and 57.16% on average, respectively, under S1D2 and S2D2 treatments. Compared with that under S0D3 treatment, breaking strength of two wheat varieties was increased by 31.26%, and 58.84% on average, respectively, under S1D3 and S2D3 treatments.

### 3.6 Effects of shading on the lignin content of wheat stems under different planting densities

**Figure 4:**
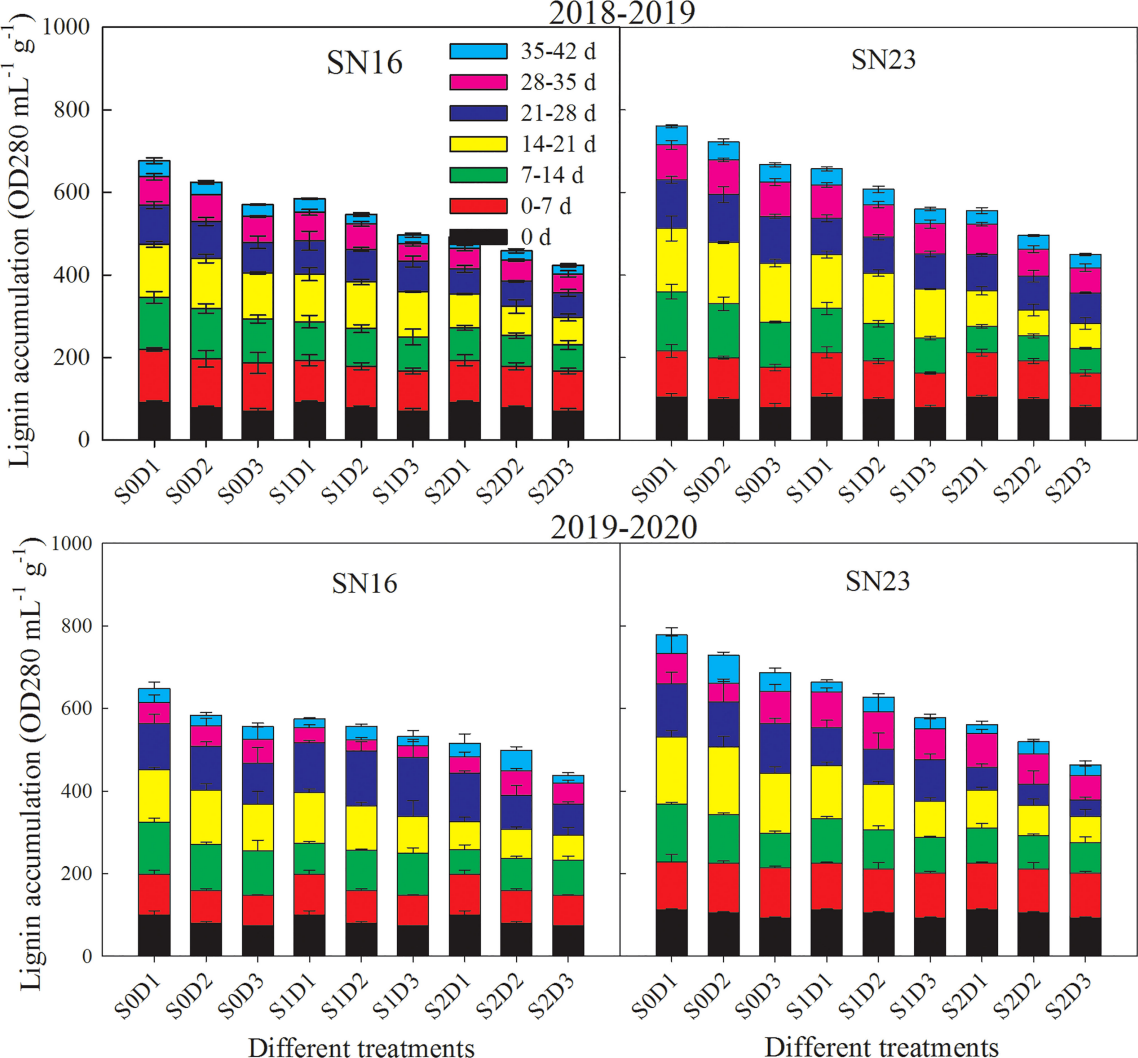
Effects of shading on the content of lignin in wheat stems under different planting densities. Error bars represent the standard deviation of the mean (n = 3).

Lignin accumulation in wheat stems gradually increased with the process of growth (**Figure 4**). The accumulation rate of lignin was faster from 0 to 28 days, while the rate tended to be relatively flat from 28 to 42 days. Compared with that of lodging sensitive wheat SN16, lignin accumulation of lodging resistant wheat SN23 was higher. Lignin accumulation of wheat stems was decreased significantly with increasing planting density. Taken 2018−2019 wheat season as an example, when planting density was increased by 75 × 10^4^ plants ha^-1^, lignin accumulation decreased by 10.14%, 11.01%, and 12.67% on average, respectively, under S0, S1, and S2 treatments. This indicates that lignin accumulation of wheat stems was more sensitive to increasing planting density under shading treatment.

Under the same planting density condition, shading significantly reduced the lignin accumulation of wheat stems, and with the extension of shading time, the decreased range became larger. As for the two wheat varieties in 2018−2019 wheat season, samples under treatments S1D1 and S2D1 had average lignin accumulation decreased by 13.56% and 27.07%, compared with those under treatment S0D1. Additionally, samples under S1D2 and S2D2 treatments had average lignin accumulation increased by 14.23% and 29.01%, compared with those under treatment S0D2. Samples under treatments S1D3 and S2D3 had average lignin accumulation increased by 14.53%, and 29.17%, compared with those under treatment S0D3.

### 3.7 Effects of shading on the content and proportion of lignin monomers in wheat stems under different planting densities

**Figure 5:**
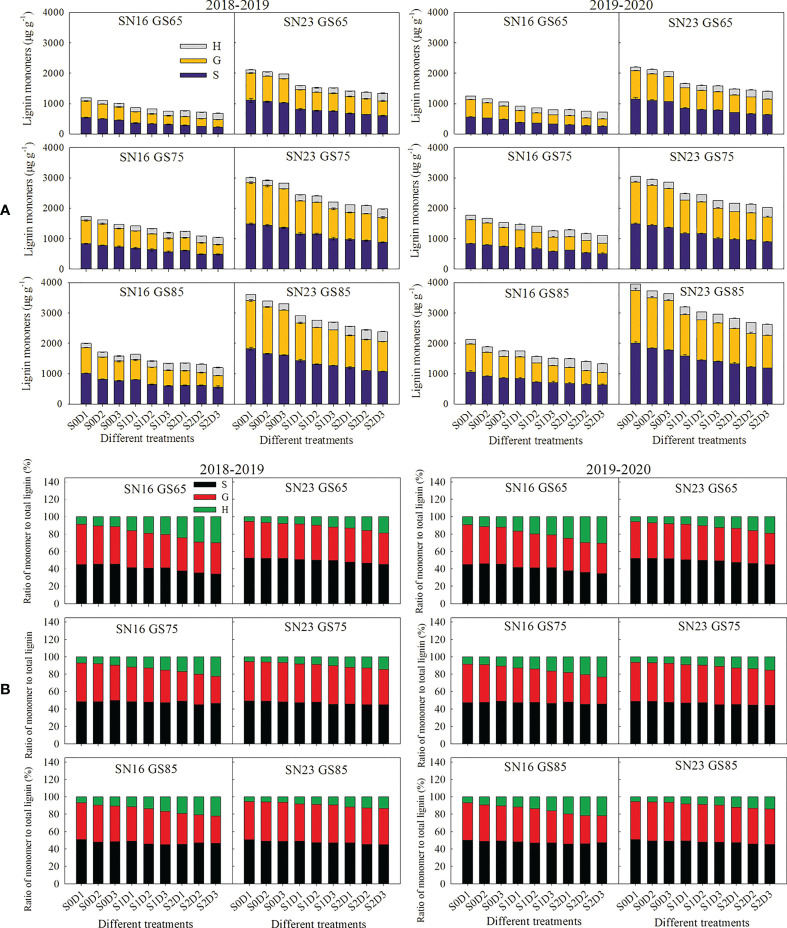
Effects of shading on the lignin monomers content **(A)** and the proportion of lignin monomers **(B)** in wheat stems under different planting densities. Error bars represent the standard deviation of the mean (n = 3).

As shown in **Figure 5**, with the advance of growth process, the content of three lignin monomers and the proportion of S monomer increased, the proportion of H monomer decreased, but the proportion of G monomer varied with different varieties and treatments. Compared with those of lodging sensitive wheat variety SN16, lodging resistant wheat variety SN23 had higher content of S, G, and H monomers, higher proportion of S and G monomers, but a lower proportion of H monomer.

With increasing planting density, the content of S and G monomers in wheat stems decreased, and the content and proportion of H monomer increased. Nevertheless, the proportion of S and G monomers varied with different wheat varieties and treatments. Taken 2018−2019 wheat season as an example, when the planting density was increased by 75 × 10^4^ plants ha^-1^, the content of S and G monomers was decreased by 58.02 μg g^-1^, and 50.92 μg g^-1^, while, the content and proportion of H monomer was enhanced by 12.26 μg g^-1^, and 13.29%.

Under the same planting density, shading reduced the content and proportion of S and G monomers in wheat stems, and increased those of H monomer. Taken 2018–2019 wheat season as an example, under shading treatment from jointing stage to heading stage (S1), the content and proportion of S monomer were decreased by 245.68 μg g^-1^, and 2.20%, respectively, the content and proportion of G monomer were decreased by 221.58 μg g^-1^, and 2.37%, but those of H monomer were increased by 36.67 μg g^-1^, and 4.57%. Under low density (D1), shading treatment reduced the proportion of S and G monomers and increased the proportion of H monomer, both of which were greater than those under high density (D2, and D3).

### 3.8 Effects of shading on the expression abundance of lignin monomers synthesis genes in wheat stems under different planting densities

**Figure 6:**
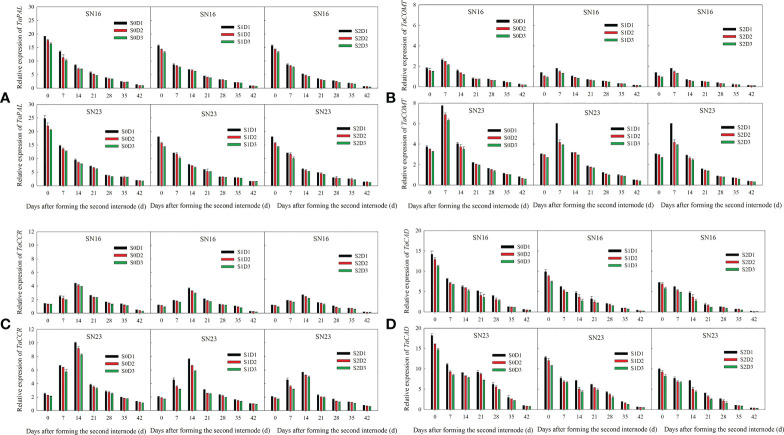
Effects of shading on the expression of lignin biosynthetic genes in wheat stems under different planting densities in 2018-2019. Error bars represent the standard deviation of the mean (n = 3). **(A)** Phenylalanine ammonialyases gene (*PAL*). **(B)** Caffeic acid O-methyl transferase gene (*COMT*). **(C)** Cinnamoyl CoA reductase gene (*CCR*). **(D)** Cinnamyl alcohol dehydrogenase gene (*CAD*).

The relative expression of *TaPAL* and *TaCAD* decreased with the progress of growth (**Figures 6A, D**), By contrast the relative expression of *TaCOMT*, and *TaCCR* first increased, then decreased, and finally peaked on day 7 and day 14, respectively (**Figures 6B, C**).

Compared with that under treatment S0D1, the relative expression of *TaPAL*, *TaCOMT*, *TaCCR*, and *TaCAD* was decreased on average by 22.24%, 25.09%, 21.80% and 30.44%, respectively, under S1D1 treatment, while 28.31%, 32.58%, 36.28% and 43.86%, under S2D1 treatment. Compared with that under S0D2 treatment, the relative expression of *TaPAL*, *TaCOMT*, *TaCCR*, and *TaCAD* was decreased on average by 19.06%, 29.07%, 4.51% and 32.42%, respectively, under S1D2 treatment, while 26.38%, 37.70%, 38.11% and 45.18%, under S2D2 treatment. Compared with that under S0D3 treatment, the relative expression of *TaPAL*, *TaCOMT*, *TaCCR*, and *TaCAD* was decreased on average by 20.26%, 29.31%, 26.89% and 34.56%, respectively, under S1D3 treatment, while 28.55%, 38.42%, 40.12% and 47.94%, under S2D3 treatment.

The above results show that shading and density significantly influenced the relative expression of genes, indicating that the relative expression was decreased with increased planting density under the same shading treatment. Under the same planting density, shading significantly reduced the relative expression of genes, and with the extension of shading time, the reduction range was increased. Moreover, lodging resistant wheat SN23 had higher relative expression of these genes than the lodging sensitive wheat SN16.

### 3.9 The relationship between breaking strength and morphological indices, lignin monomers composition of wheat stems

Correlation analysis shows that the breaking strength of wheat stems was significantly positively correlated with stem diameter, wall thickness, filling degree, lignin content, the contents and proportions of monomers S and G (*P* < 0.01), and negatively correlated with length of the basal second internode, height of center of gravity, H monomer content and proportion (*P* < 0.01) (**Figure S4**).

To further analyze the primary and secondary effects of stem morphological indices and lignin monomers composition on the breaking strength of stems under shading treatment, this study analyzed the relationship between the breaking strength and the stem diameter, wall thickness, filling degree, the length of the second basal internode, CG height, lignin content, the contents of monomers S, G, and H, and the proportions of monomers S, G, and H (recorded as X1, X2, X3, X4, X5, X6, X7, X8, X9, X10, X11, and X12, respectively) through a stepwise regression analysis (**Table 1**). When P < 0.05, the independent variable entered the stepwise regression equation. Conversely, when P > 0.05, the independent variable did not enter the regression equation. According to this principle, X2, X3, X4, X6, X7, X9, and X12 entered the equation, and the partial correlation coefficients of the seven variables were in the order of X3 (0.734) > X2 (0.656) > X9 (-0.580) > X6 (-0.422) > X7 (0.407) > X4 (0.284) > X12 (-0.203). This result indicates that stem filling degree, wall thickness, H monomer content, lignin content, S monomer content, length of the basal second basal internode, and H monomer proportion are the main factors affecting the breaking strength of stems in wheat in response to shading stress. Others are the secondary factors.

**Table 1:** Stepwise regression analysis between wheat stem morphological indices, lignin monomers composition and breaking strength.

Indices	Stepwise regression equation	Variables	Partial correlation coefficient	P value
Morphological characteristics	Y=-13.35+10.63X2+0.31X3+0.51X4	Wall thickness (X2)	0.6557	0.0001
		Filling degree (X3)	0.7335	0.0001
		Second internode length (X4)	0.2843	0.0031
Lignin and monomers composition	Y=33.58-0.03X6+0.01X7-0.07X9-0.22X12	Lignin content (X6)	-0.4227	0.0001
		S monomer content (X7)	0.4069	0.0001
		H monomer content (X9)	-0.5804	0.0001
		H monomer proportion (X12)	-0.2028	0.0380

### 3.10 Variance analysis of the morphological indices, lignin monomers composition and bending resistance of wheat stems

It was indicated that year had no significant effects on the breaking strength, stem diameter, wall thickness, filling degree, CG height and S monomer content of stems, but had significant effects on the length of the second basal internode, the content and proportion of G and H monomers (**Table 2**). Variety, shading and planting density had significant effects on the breaking strength, morphological indices and lignin monomers composition of wheat stems. Shading and planting density interacted significantly on the wall thickness and the proportion of lignin monomers of wheat stems (*P* < 0.01).

**Table 2:** Analysis of variance of the effects of year (Y), cultivar (C), shading (N) and density (D) on breaking strength, morphological characteristics, and lignin monomers composition of stems in wheat at milk stage.

Source of variation	BS	SD	WT	SFD	LSI	HCG	LC	S content	G content	H content	S proportion	G proportion	H proportion
Year (Y)	ns	ns	ns	ns	**	ns	**	ns	*	**	**	**	**
Cultivar (C)	**	**	**	**	**	**	**	**	**	**	**	**	**
Shading (S)	**	**	**	**	**	**	**	**	**	**	**	**	**
Density (D)	**	**	**	**	**	**	**	**	**	**	**	**	**
C×S×	**	**	ns	**	ns	**	**	**	**	ns	**	**	**
C×D×	*	ns	ns	ns	ns	ns	*	ns	ns	ns	**	**	**
S×D×	ns	**	ns	ns	ns	**	ns	ns	ns	ns	**	**	**
C×S×D×	ns	**	ns	ns	ns	ns	ns	ns	ns	ns	**	**	**

BS, breaking strength; SD, Stem diameter; WT, Wall thickness; SFD, Stem filling degree; LSI, Length of the second internode; HCG, Height of center of gravity; LC, Lignin content. ns, *and ** represent no significant, significant at 0.05 and 0.01 levels, respectively.

## 4 Discussion

### 4.1 Effects of shading on the breaking strength and morphological characteristics in wheat stems under different planting densities

Shading and density significantly influenced the interception rate of PAR of two wheat varieties, shown as S0 > S1 > S2 and D3 > D2 > D1 (**Figure S3**). Moreover, the breaking strength was markedly affected by planting density and shading (**Table 1**). The breaking strength of the second basal internode in two wheat varieties was decreased with increased planting density at different stages. The breaking strength in wheat stems showed a larger decline range. At high density of 300 × 10^4^ plants ha^-1^, shading at the jointing stage reduced the stem breaking strength of two wheat varieties more significantly than that at low density (150 × 10^4^ plants ha^-1^), and medium density (225 × 10^4^ plants ha^-1^). The results indicated that the breaking strength of wheat stems was more sensitive to shading stress under high density. The formation of breaking strength is mainly affected by morphological characteristics and structural component content of crop stems (Zhang R. et al., 2020; Guo et al., 2021).

The morphological characteristics (plant height, CG height, internode length, stem diameter, wall thickness, filling degree, etc.) of crop stems are influenced by shading stress and planting density. As the density increased, the plant height of maize first increased and then decreased. The length of basal internodes (especially the third basal internodes) significantly increased. Conversely, the stem diameter, cell wall thickness mechanical tissue, mechanical cell layer, and cortex decreased. As a result, the lodging rate of maize gradually increased (Sher et al., 2018). Moreover, the plant height, and the CG height of wheat increased. The filling degree of stems were decreased, resulting in the decline of stem breaking strength. Consistent with that, our research showed that with the increase of planting density, the internode length, spike length, and the plant height were increased and the CG height moved up (**Figure 1A**). When the planting density was increased by 75 × 10^4^ plants ha^-1^, the plant height and the CG height increased by 1.82 cm and 1.11 cm, respectively under shading treatment from the jointing stage to the heading stage, with 1.57 cm and 0.86 cm, respectively, to offset the negative effects partially of shading stress (Lyu et al., 2021).

It was found that shading treatment from the jointing stage to the heading stage decreased the plant height of rice, but had no significant difference from the normal light treatment. Besides, the lengths of the first and second basal internodes increased in response to shading treatment. The spike length, the lengths of the first and the second internodes from the spike, the stem diameter and wall thickness of the second basal internode showed the opposite regularity (Zhang, W. J. et al., 2020). Wu et al. (2017) reported that the plant height and CG height increased in response to shading stress, however, the stem diameter and wall thickness were reduced. The results of this study showed that shading stress at the jointing stage significantly increased the lengths of the second to the fifth basal internodes (especially the fourth internode), and the spike length, resulting in higher plant height, and CG height of wheat (**Figure 1A**). However, the stem diameter and wall thickness of the second internode were reduced due to shading stress (**Figures 1B, C**). With the extension of shading time, the above indicators increased or decreased much more. Liu et al. (2018) found that shading significantly increased plant height, but decreased stem diameter, which leading to higher lodging risk of soybean. However, previous researches demonstrated that the effects of shading stress on soybean varied with different cultivars (Yao et al., 2017). The above results showed that shading had different effects on plant height, CG height and internode length, mainly due to different shading periods and crop types (**Figure 1A**), but had the same effects on crop stem diameter and wall thickness (**Figures 1B, C**). According to comprehensive correlation analysis (**Figure S4**) and stepwise regression analysis (**Table 1**), the filling degree, wall thickness and length of the second basal internode are the main factors affecting the breaking strength of wheat stems in response to shading stress, and others are secondary factors.

At planting density of 150 × 10^4^ plants ha^-1^, shading from the jointing to the heading stages increased the plant height and CG height of two wheat varieties by 8.88%, and 16.17% on average. At planting density of 225 × 10^4^ plants ha^-1^, these were 7.17%, and 6.74%. Besides, when planting density was 300 × 10^4^ plants ha^-1^, these were 4.51%, and 3.55% (**Figure 1A**). These results indicated that under the condition of high density, the increase in plant height and CG height (**Figure 1A**) of wheat were lower than those at low and medium density. This may be because of shade avoidance of wheat under population densification. By extension, shading signals are perceived by the phytochrome photoreceptors, phytochrome B (phyB) and phyA, which play antagonistic roles in stem elongation. Lowering the R:FR to shading stress deactivates phyB, resulting in the internode elongation promotion. By contrast, when the planting density is high, R:FR is very low under shading conditions. Thus, phyA accumulates and is strongly activated under very low R:FR to prevent excessive internode elongation of wheat (Molina-Contreras et al., 2019; Paulišić et al., 2021). Therefore, shading promoted the elongation of wheat internodes, and its elongation decreased with an increase in the planting density.

### 4.2 Effects of shading on the lignin metabolism in wheat stems under different planting densities

Welton (1928) first proposed that lodging was caused by the lack of lignin in wheat stems. Lignin could support and prevent the stems from lodging. It was also exhibited that the content of lignin in pex1 semi dwarf mutant was increased, and the lodging resistance of rice was enhanced. The breaking strength of wheat stems is determined by the vascular system and mechanical tissues, and lignin provides rigidity for stems (Khobra et al., 2019). The rigidity strength of stems is mainly composed by two parts: ① oxidative covalent crosslinking strength of lignin polymer chains and cell wall polysaccharides; ② filling degree of lignin polymer chains in vascular units such as cellulose macrofibrils and hemicellulose (Ragauskas et al., 2014; Anderson et al., 2015; Balakshin et al., 2020). Lignin plays a decisive role as a “binder” in the formation of breaking and pressure strength of stems. It can be seen that the synthesis and accumulation of lignin is the basis for determining the rigid breaking resistance of stems. This study indicated that the lignin content of the second basal internode in wheat increased gradually after the jointing stage. The accumulation rate of lignin became faster from 0 to 28 days after the jointing stage. Lignin was also enhanced from 28 to 42 days after the jointing stage, but the rate tended to be stable (**Figure 4**). Shading apparently reduced lignin accumulation, and with the extension of shading time, lignin accumulation in wheat stems was significantly decreased. At the planting density of 150 × 10^4^ plants ha^-1^, S1 and S2 treatments decreased the lignin accumulation by 13.56%, and 27.07%, respectively at 42 days after jointing. At the planting density of 225 × 10^4^ plants ha^-1^, S1 and S2 treatments decreased the lignin accumulation by 14.23%, and 29.01%, respectively. Moreover, at the planting density of 300 × 10^4^ plants ha^-1^, S1 and S2 treatments decreased the lignin accumulation by 14.53%, and 29.17%, respectively. It was indicated that lignin accumulation in wheat stems was more sensitive to shading under high density conditions.

*TaPAL*, *TaCOMT*, *TaCCR*, and *TaCAD* are key enzyme genes of carbon flow distribution in wheat lignin synthesis pathway. Their expression abundance significantly influenced the synthesis and accumulation of lignin (Ma, 2007; Ma, 2010; Levy et al., 2018). The results of this study showed that the relative expression of these genes of lodging resistant wheat SN23 was significantly higher than that of lodging sensitive wheat SN16 (**Figure 6**), and the stem lignin content of SN23 was higher than that of SN16 (**Figures 4**, **5**), indicating that SN23 showed a comparatively greater lodging resistance in response to shade stress and high planting density. Shading and planting density significantly affected the expression of key enzyme genes in the lignin synthesis pathway of wheat stems. With the increasing planting density, the expression of *TaPAL*, *TaCOMT*, *TaCCR* and *TaCAD* in wheat stems was decreased. Moreover, it was also reduced in response to shading stress, which was consistent with the change of lignin accumulation and breaking strength of stems. At high density, the reduction range of the expression of *TaPAL*, *TaCOMT*, *TaCCR* and *TaCAD* in wheat stems suffered from shading stress was larger than that at low density and medium density. The results suggested that the expression of those genes was much more sensitive to shading stress at high density, which was related to lignin accumulation and breaking strength of stems.

TaPAL is the key enzyme in the carbon flow distribution in the lignin pathway, and its downregulation mainly decreases the G monomer (Feduraev et al., 2021). TaCOMT is the key enzyme in the formation of G monomer switched into S monomer and free radicals from carbon sources, while its downregulation reduces the proportion of S and H monomers, but increases the proportion of G monomer (Wang et al., 2018). TaCCR and TaCAD are enzymes that catalyze the last two steps of lignin monomers synthesis, respectively (Ma, 2007; Ma, 2010). When CCR was downregulated, there was a slight decrease in lignin content, but significant changes in lignin composition. The ratio of H monomer was dramatically reduced and the S/G ratio was slightly enhanced (Tamasloukht et al., 2011). Downregulation of *CAD* would result in lower content of S monomer, higher content of G and H monomers and lower S/G ratio (Trabucco et al., 2013). The results of this study showed that shading decreased PAR interception rate of two wheat varieties, reduced the content and proportion of S and G monomers, but increased the content and proportion of H monomer, which was due to a combination of factors, including downregulation of *TaPAL*, *TaCOMT*, *TaCCR*, and *TaCAD* genes under shading stress. Under the condition of shading from jointing stage to heading stage, the content and proportion of S monomer were reduced by 245.68 μg g^-1^ and 2.20%, while those of G monomer were decreased by 221.58 μg g^-1^ and 2.37%, respectively. By contrary, the content and proportion of H monomer were enhanced by 36.67 μg g^-1^ and 4.57%, respectively. Moreover, under the condition of shading from jointing stage to maturity stage, the content and proportion of S monomer were reduced by 375.96 μg g^-1^ and 4.55%, while those of G monomer were decreased by 357.64 μg g^-1^ and 6.07%, respectively. The content and proportion of H monomer were enhanced by 95.12 μg g^-1^ and 10.63%, respectively. These results indicated that the changed range of lignin monomers in wheat stems was increased with the extension of shading time (**Figure 5**). According to comprehensive correlation analysis (**Figure S4**) and stepwise regression analysis (**Table 1**), the content and proportion of H monomer, lignin content and S monomer content in wheat stems are the main factors influencing the breaking strength of wheat stems. Among these factors, the content and proportion of H monomer were negative factors, whereas lignin content and S monomer content were positive factors. The above results demonstrated that under the condition of shading at jointing stage, the expression of *TaPAL*, *TaCOMT*, *TaCCR*, and *TaCAD* in wheat stems decreased (**Figure 6**), thereby, the lignin accumulation and monomers composition of wheat stems were changed. Lignin content and S monomer at 42 days after jointing were decreased by 18.48%, and 296.49 μg g^-1^, respectively. The content and proportion of H monomer were enhanced by 69.82 μg g^-1^ and 8.37%, respectively (**Figure 5**).

## 5 Conclusions

**Figure 7:**
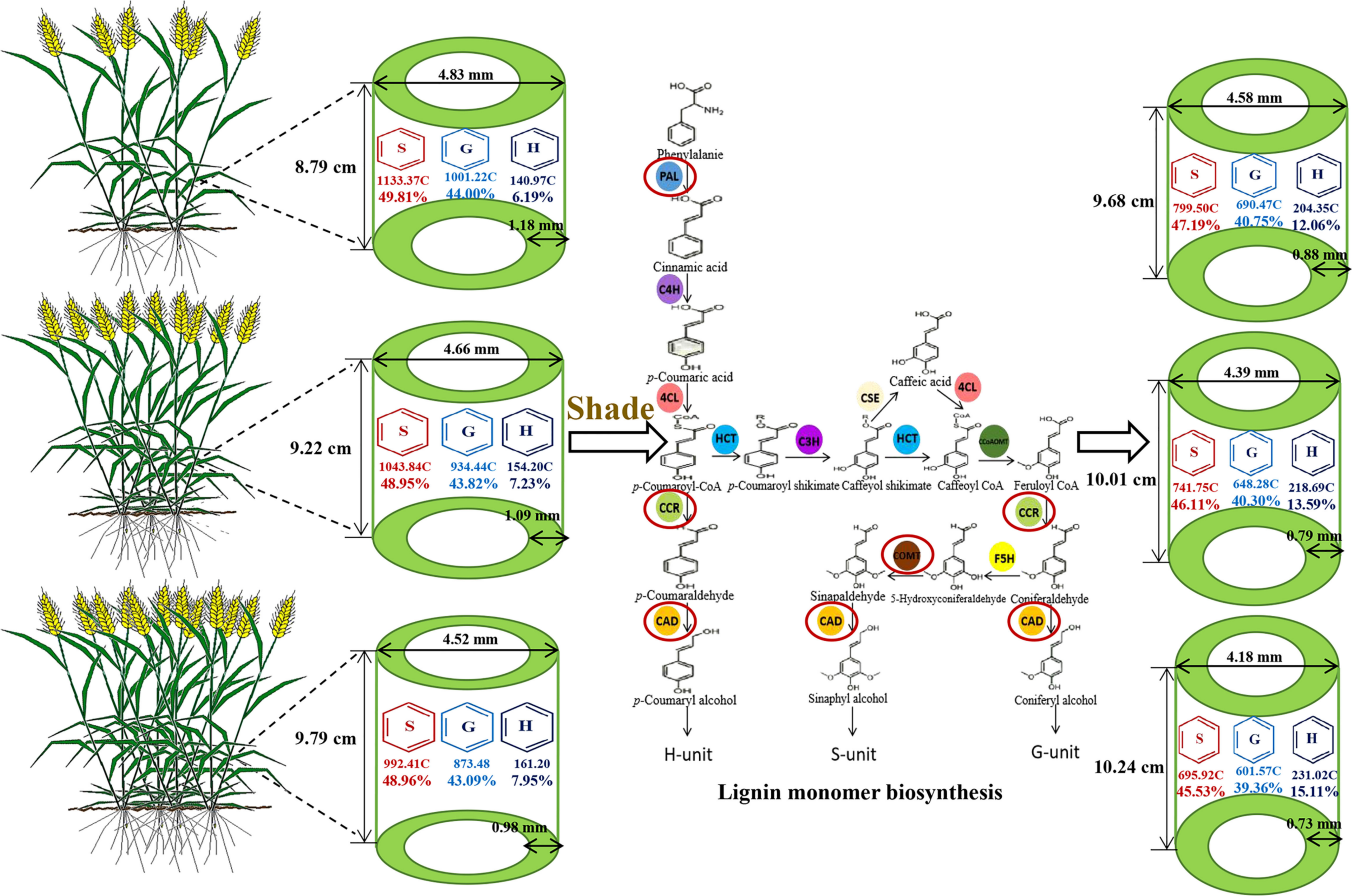
Pattern graph of shading affecting stem morphological characteristics and lignin metabolism of wheat under different planting densities. S, syringyl; G, guaiacyl; H, *p*-hydroxyphenyl; C, concentration unit (μg g^-1^). PAL, phenylalanine ammonia-lyase; C4H, cinnamate 4-hydroxylase; 4CL, 4-hydroxycinnamate:CoA ligase; HCT, *p*-hydroxycinnamoyl-CoA shikimate/quinate hydroxycinnamoyl transferase; C3H, coumarate 3-hydroxylase; CSE, caffeoyl shikimate esterase; CCoAOMT, caffeoyl CoA o-methyl transferase; F5H, ferulate/coniferaldehyde 5-hydroxylase; CCR, cinnamoyl CoA reductase; CAD, cinnamyl alcohol dehydrogenase.

The decreased filling degree, wall thickness, lignin content, and S monomer content in wheat stems, the increased content and proportion of H monomer, and the increased length of the second basal internode play pivotal roles in decreasing the breaking strength of wheat stems in response to shading stress. Under shading stress and high density, the limited photosynthetic carbon source flowing to the lignin synthesis pathway hinders the expression of *TaPAL*, *TaCOMT*, *TaCCR* and *TaCAD*. As a result, the lignin synthesis and its distribution proportion in S, G, and H monomers were altered. The lignin accumulation and the S monomer content decreased. Additionally, the content and proportion of H monomer were increased, which led to low breaking strength (**Figure 7**).

## Data availability statement

The original contributions presented in the study are included in the article/**Supplementary Material**. Further inquiries can be directed to the corresponding authors.

## Author contributions

YLu and YC: Software, Methodology, Investigation, Data Curation, Writing – Original Draft. CL: Methodology, Validation, Data curation. YW, HC and MJ: Methodology, Data curation. ZW and YLi: Conceptualization, Funding acquisition, Writing – review and editing, Validation, Supervision. All authors contributed to the article and approved the submitted version.

## Funding

The work was supported by the National Natural Science Foundation of China (NO. 32101834, 32172117), the Natural Science Foundation of Shandong Province (NO. ZR2020QC106), Post-doctoral Science Foundation of China (2022M711968), the Shandong Mount Tai Program for Industrial Leading Talents (NO. LJNY2015001), the National Key Research and Development Program of China (NO. 2017YFD0301001, 2016YFD0300403).

## Conflict of interest

The authors declare that the research was conducted in the absence of any commercial or financial relationships that could be construed as a potential conflict of interest.

## Publisher’s note

All claims expressed in this article are solely those of the authors and do not necessarily represent those of their affiliated organizations, or those of the publisher, the editors and the reviewers. Any product that may be evaluated in this article, or claim that may be made by its manufacturer, is not guaranteed or endorsed by the publisher.
