# Supplementary material for: Shading decreases lodging resistance of wheat under different planting densities by altering lignin monomer composition of stems
Source: Front Plant Sci. 2022 Nov 17;13:1056193. doi: 10.3389/fpls.2022.1056193 (PMC9714359; doi:10.3389/fpls.2022.1056193)
Supplement: Supplementary file 1 [file DataSheet_1.docx]

Supplementary Material

# Supplementary Figures, Tables and References

## Supplementary Table

**Table S1** The detailed primers of the key enzyme genes in lignin biosynthesis pathway.

| Gene name | Gene ID | Forward primer (5’-3’) | Reverse primer((5’-3’) |
| --- | --- | --- | --- |
| *TaPAL4* | TraesCS6A02G222800 | CATCTTGGAGGGAAGCTCATAC | GACTTGGTGGCAAATCGAATAAC |
| *TaCCR2* | TraesCS5D02G232400 | CGTGATGGTGCTGAAGAAAC | CGATCATCGAAGCCGATACA |
| *TaCAD4* | TraesCS6D02G162800 | AAAAGCAGAGGGAGAAAGAGC | AACGGAAACAGGTCACAAACAT |
| *TaCOMT1* | TraesCS3B02G612000 | GATCCATGACAACGAGTCTACC | CGAATCAATCGACGACACAAAC |
| *β-Actin* | TraesCS1A02G274400 | GGGACCTCACGGATAATCTAATG | CGTAAGCGAGCTTCTCCTTTAT |

## Supplementary Figures


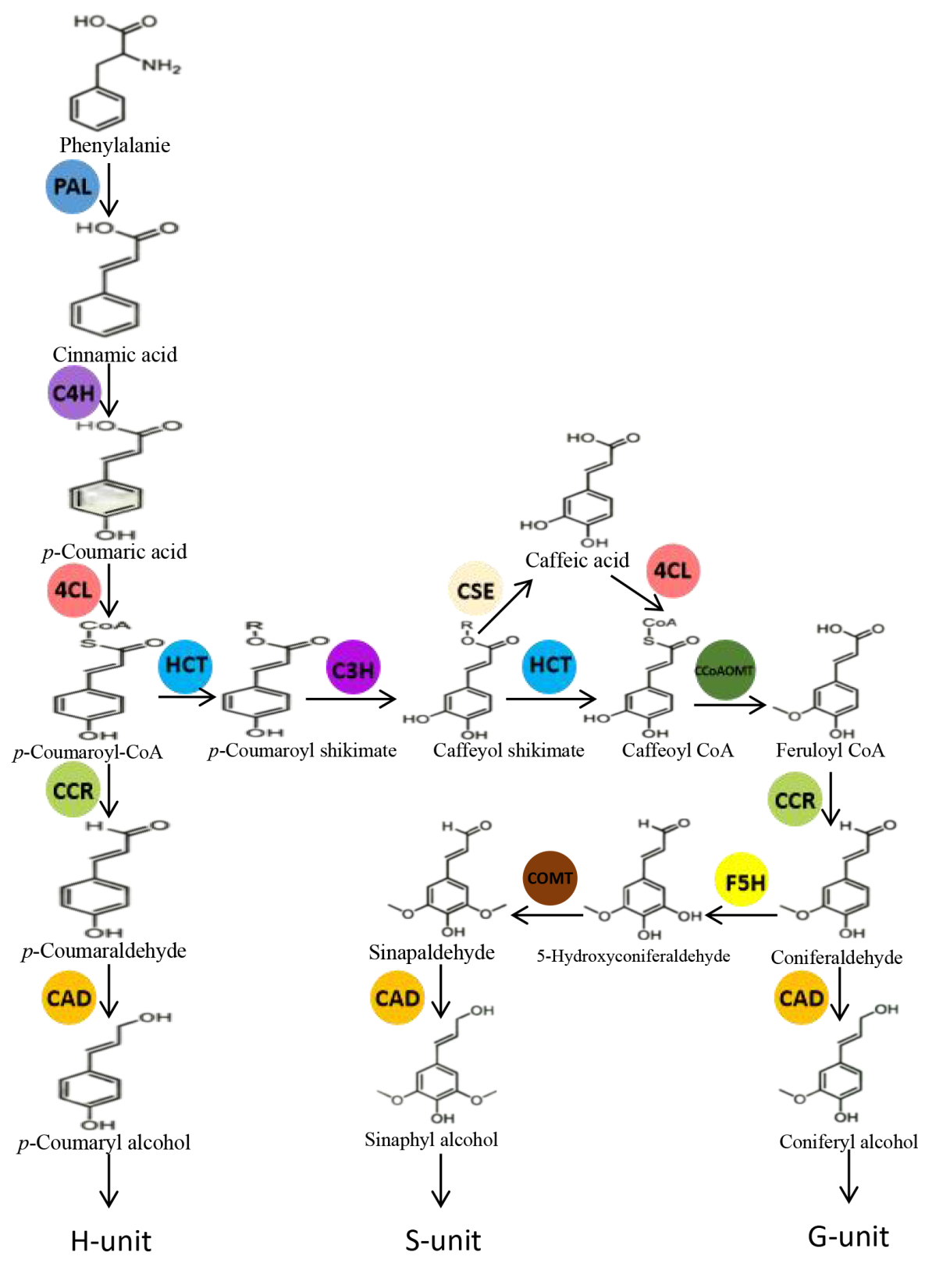


**Supplementary Figure 1.** **The pathway of lignin biosynthesis (Rinaldi et al., 2016).**

S, syringyl；G, guaiacyl; H, *p*-hydroxyphenyl; PAL, phenylalanine ammonia-lyase; C4H, cinnamate 4-hydroxylase; 4CL, 4-hydroxycinnamate:CoA ligase; HCT, *p*-hydroxycinnamoyl-CoA shikimate/quinate hydroxycinnamoyl transferase; C3H, coumarate 3-hydroxylase; CSE, caffeoyl shikimate esterase; CCoAOMT, caffeoyl CoA o-methyl transferase; F5H, ferulate/coniferaldehyde 5-hydroxylase; COMT, caffeate/5-hydroxyferulate 3-O-methyltransferase; CCR, cinnamoyl CoA reductase; CAD, cinnamyl alcohol dehydrogenase.


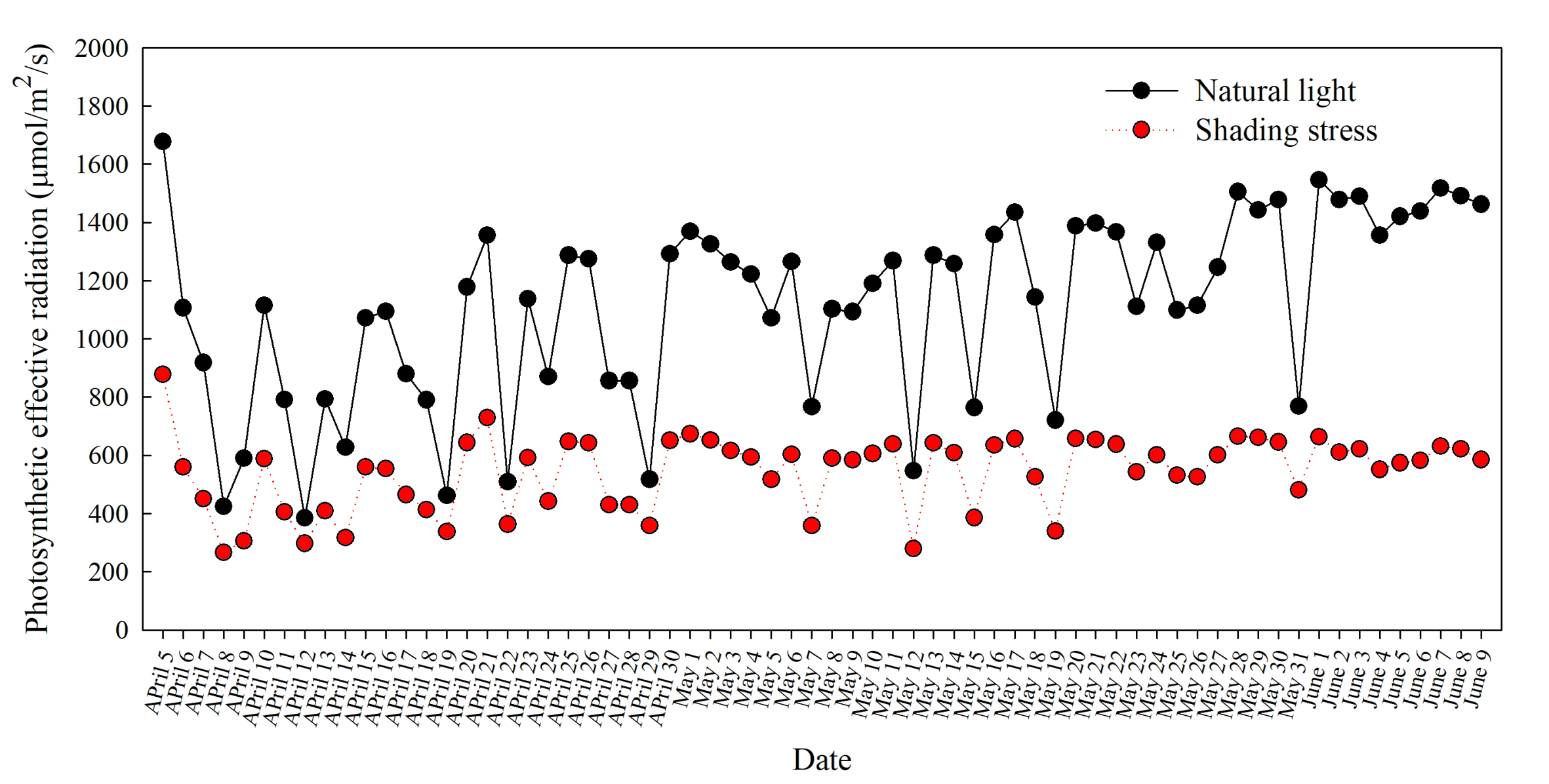


**Supplementary Figure 2. Photosynthetic effective radiation (PAR) intensity from jointing stage to maturity stage of wheat under natural light and shading stress treatments.**

**
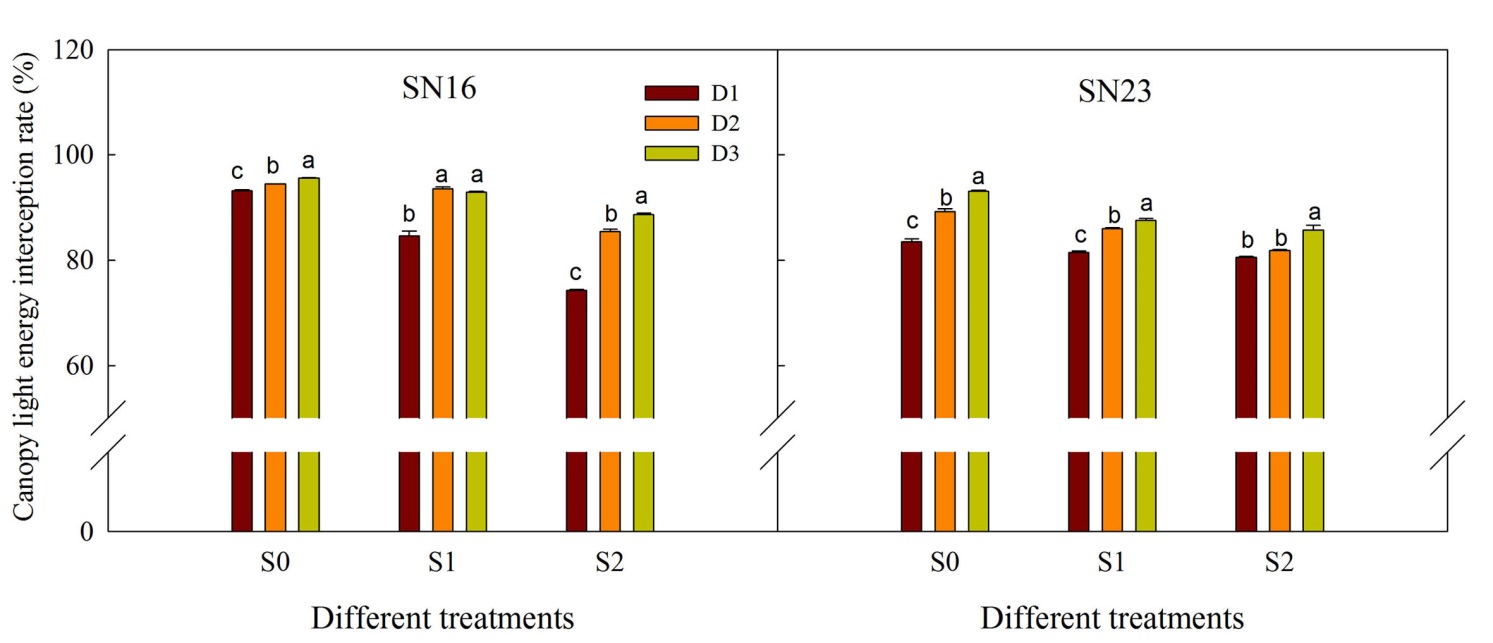
**

**Supplementary Figure 3. Effects of shading on canopy light energy interception rate in different winter wheat populations at anthesis stage.** SN16, Shannong16; SN23, Shannong23; S0, natural light; S1, shading from jointing to heading stage; S2, shading from jointing to maturity stage; D1, 150 × 10^4^ plants ha^-1^; D2, 225 × 10^4^ plants ha^-1^; D3, 300 × 10^4^ plants ha^-1^. Error bars represent the standard deviation of the mean (n = 10). Different lowercase letters indicate significant differences among treatments at the 0.05 level. The same as below.


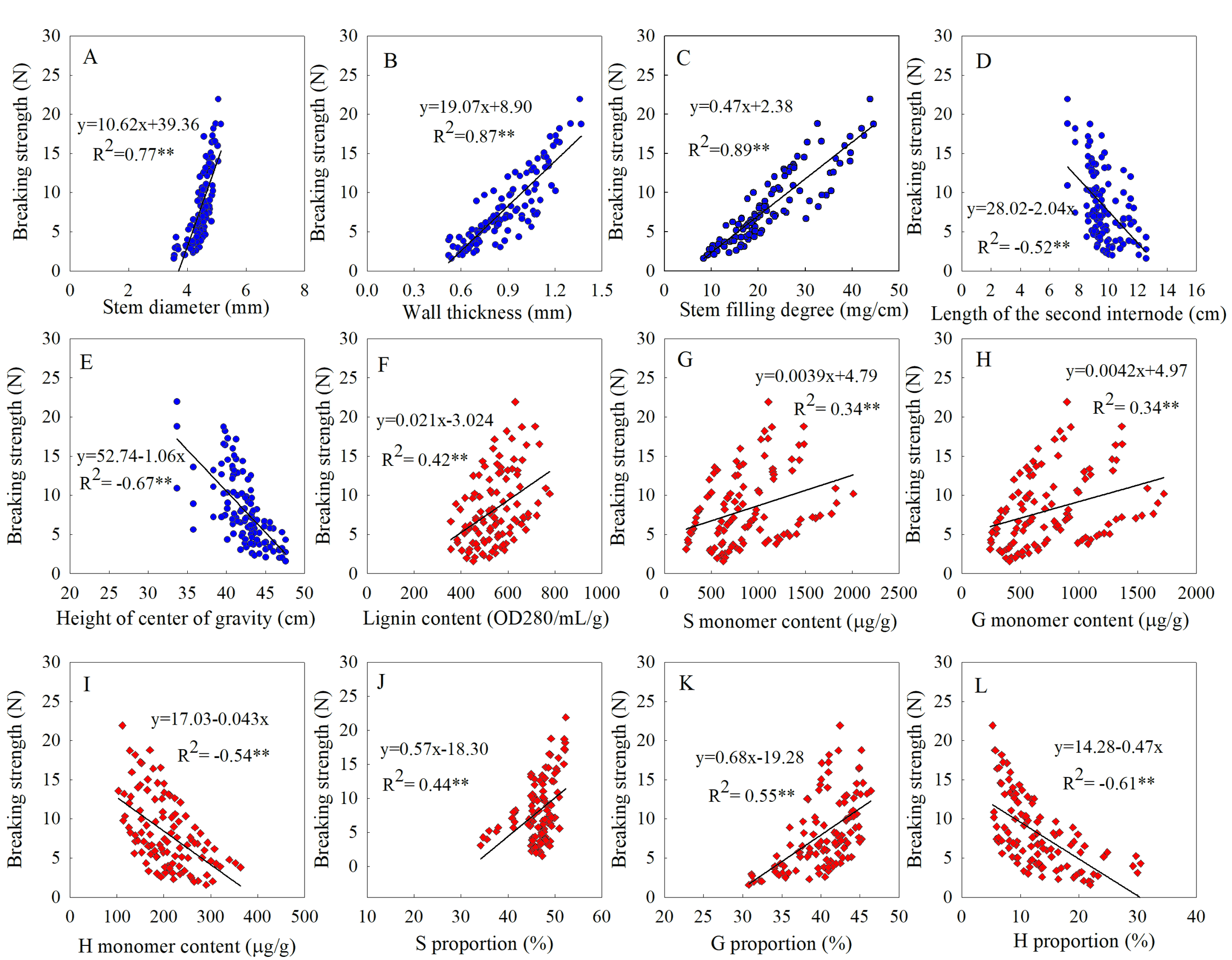


**Supplementary Figure 4. Relationship between breaking strength and morphological indexes and lignin monomers composition of wheat stems.**

## Supplementary Reference

Rinaldi, R., Jastrzebski, R., Clough, M. T., Ralph, J., Kennema, M., Bruijnincx, P. C. A., et al. (2016). Paving the way for lignin valorisation: recent advances in bioengineering, biorefining and catalysis. Angew. Chem. Int. Ed. 55, 8164–8215. doi: 10.1002/anie.201510351
